# Emotional Dysregulation and Emotional Eating in Hospitalized Adults with Obesity: The Mediating Role of Worry and Rumination

**DOI:** 10.3390/jcm14113871

**Published:** 2025-05-30

**Authors:** Anna Guerrini Usubini, Sara Ducale, Adele Bondesan, Francesca Frigerio, Gabriella Tringali, Mauro Cornacchia, Ferruccio Nibbio, Gianluca Castelnuovo, Alessandro Sartorio

**Affiliations:** 1 Experimental Laboratory for Auxo-Endocrinological Research, Istituto Auxologico Italiano, Istituto di Ricovero e Cura a Carattere Scientifico IRCCS, 28824 Piancavallo-Verbania, Italy; a.bondesan@auxologico.it (A.B.); f.frigerio@auxologico.it (F.F.); g.tringali@auxologico.it (G.T.); sartorio@auxologico.it (A.S.); 2 Psychology Research Laboratory, Istituto Auxologico Italiano, Istituto di Ricovero e Cura a Carattere Scientifico IRCCS, 28824 Piancavallo-Verbania, Italy; s.ducale@auxologico.it (S.D.); gianluca.castelnuovo@auxologico.it (G.C.); 3 Division of Pneumology, Istituto Auxologico Italiano, Istituto di Ricovero e Cura a Carattere Scientifico IRCCS, 28824 Piancavallo-Verbania, Italy; m.cornacchia@auxologico.it; 4 Division of Cardiology, Istituto Auxologico Italiano, Istituto di Ricovero e Cura a Carattere Scientifico IRCCS, 28824 Piancavallo-Verbania, Italy; f.nibbio@auxologico.it; 5 Department of Psychology, Catholic University of Milan, 20123 Milan, Italy

**Keywords:** obesity, worry, rumination, emotional dysregulation, emotional eating

## Abstract

**Background:** Emotional dysregulation has been strongly linked to maladaptive eating behaviors in obesity. Worry and rumination are frequently implicated in emotional dysregulation and may serve as pathways linking emotional regulation difficulties to emotional eating. The current study examines the mediating role of worry and rumination in the relationship between emotional dysregulation and emotional eating among individuals with obesity. **Methods**: Ninety hospitalized Italian adults were involved in the study with 53 obese males, 37 obese females, mean age ± SD: 50.1 + 10.9 years; mean body mass index: 46.4 ± 9.4 kg/m^2^. To assess worry, rumination, emotion dysregulation, and emotional eating, the participants were asked to fill in, respectively, the following questionnaires: The Penn State Worry Questionnaire; The Ruminative Response Scale; The Anger Rumination Scale; The Difficulties in Emotion Regulation Scale; Emotional Eating subscale of the Dutch Eating Behavior Questionnaire. Three mediation models were tested to examine the relationships between difficulties in emotional regulation as a predictor, worry and rumination as mediators separately, and emotional eating as the dependent variable. **Results**: The mediation analyses revealed significant indirect effects across all models, suggesting the presence of mediation effects of worry and rumination in the relationships between emotional dysregulation and emotional eating. **Conclusions**: These findings highlight the critical mediating role of worry and rumination that drive the observed relationships between emotional dysregulation and emotional eating. This study contributes to a deeper understanding of the cognitive-emotional mechanisms involved in emotional eating in individuals with obesity. Such results can contribute to developing targeted interventions aimed at improving emotional regulation and reducing maladaptive eating behaviors.

## 1. Introduction

Obesity is a complex and chronic disease characterized by excessive body fat accumulation that poses significant health risks [1]. Body mass index (BMI) is commonly used to classify obesity, with a BMI of 30 or higher indicating obesity. According to the Centers for Disease Control and Prevention (CDC), more than two in five U.S. adults suffer from obesity, highlighting its prevalence and public health impact. According to the Italian Health Examination Survey (HES) 2023, the prevalence of obesity among adults aged 35–74 years was 23% in men and 25% in women [2]. Obesity is associated with an increased risk of various health conditions, including type 2 diabetes and cardiovascular diseases. In addition, psychological distress, such as anxiety and depression [3], and social disadvantages (i.e., low socioeconomic status [4] are associated with higher rates of obesity.

Several studies highlighted the critical role of emotional dysregulation—the inability to manage and respond to emotional experiences appropriately [5]—in the development and persistence of obesity [6]. Emotional dysregulation, characterized by difficulties in understanding, accepting, and managing emotions, as well as limited access to adaptive emotion regulation strategies [7] has been associated with the use of specific strategies (e.g., avoidance, rumination, worry, emotion suppression) to dysfunctionally regulate emotions [8,9]. In addition, emotional dysregulation has been reported to underlie the etiological and maintenance mechanisms in a wide range of psychological disorders, including eating disorders [10,11]. Emotional dysregulation showed a significant relationship with maladaptive eating behaviors, such as emotional eating and binge eating disorder (BED), a common comorbid condition with obesity [12]. Emotional eating has been defined as eating in response to negative emotions such as anxiety, anger, or sadness, in order to reduce emotional distress [13,14]. BED is an eating disorder characterized by recurrent episodes of eating large quantities of food, often very quickly and to the point of discomfort, accompanied by a feeling of loss of control during the binge, followed by distress, guilt, or shame, but without regular compensatory behaviors (e.g., purging, excessive exercise) [15]. A meta-analysis encompassing 23 studies revealed a medium positive connection between difficulties in regulating emotions and disordered eating behaviors, including emotional eating. The analysis also indicated that this association was more pronounced in females [16]. In 2024, an observational study examined the impact of negative emotional dysregulation on the outcomes of bariatric surgery in patients with severe obesity. The study found that patients with higher levels of emotional dysregulation had less favorable weight loss outcomes one year post-surgery, highlighting the importance of addressing emotional regulation in pre- and post-operative care [17].

In the literature, the relationship between obesity and emotional eating is well-documented. Vasileiou and Abbott [18] found that individuals who were overweight and obese showed higher scores in emotional eating than individuals with a normal weight. Similarly, a study by Geliebter and Aversa [19] reported that individuals with obesity were more likely brought to eat in response to negative emotions, such as stress and sadness, compared to their normal-weight counterparts. A recent study examining the prevalence of emotional eating in overweight and obese individuals found that the prevalence of emotional eating in such individuals was 44.9%, indicating that nearly half of this population engages in emotional eating behaviors [20].

Two maladaptive cognitive-emotional processes—worry and rumination—are frequently implicated in emotional dysregulation. They represent two forms of negative repetitive thinking and may serve as pathways linking emotional regulation difficulties to emotional eating [21,22]. Worry is defined as a chain of thoughts and images, predominantly negative and relatively uncontrollable, that are perceived as an attempt to anticipate and prepare for future threats. Rumination, on the other hand, refers to continuously thinking about the same thoughts, which are often sad or dark and are generally focused on past events. It involves a repetitive and passive focus on the causes and consequences of one’s distress without active problem solving, leading to prolonged emotional suffering [21]. Rumination is composed of anger rumination and depressive rumination [23]. Depressive rumination is characterized by persistent, passive, and self-focused thoughts about one’s distress, failures, and perceived inadequacies [21]. It is strongly linked to feelings of sadness, hopelessness, and impaired problem-solving abilities, playing a crucial role in the onset and maintenance of depression [24]. In contrast, angry rumination involves repetitive thoughts about perceived injustices and provocations, leading to prolonged anger and increased aggression [25]. Both worry and rumination have been linked to emotional distress and dysfunctional coping mechanisms, including overeating [26,27,28].

Despite the well-documented relationship between emotional dysregulation and emotional eating, limited research has explored the specific role of worry and rumination in this association, particularly among adults with obesity in the Italian context. In addition, examining worry and rumination as potential mediators could provide a more in-depth understanding of how emotional and cognitive dysregulation can influence eating behaviors and weight outcomes.

The current cross-sectional study examined the mediating role of worry and rumination in the relationships between emotional dysregulation and emotional eating among hospitalized individuals with obesity. Specifically, we investigated whether difficulties in emotional regulation predicted emotional eating, with worry and rumination serving as separate mediators.

## 2. Materials and Methods

### 2.1. Participants and Procedures

Ninety hospitalized Italian adults with obesity were involved in the study during a 3-week body weight reduction program (53 males, 37 females, mean age ± SD: 50.1 + 10.9 years; mean body mass index, BMI: 46.4 ± 9.4 kg/m^2^). The participants were consecutively recruited at Istituto Auxologico Italiano, IRCCS, Piancavallo-Verbania, a third-level clinical center for multidisciplinary rehabilitation of obesity. The inclusion criteria were as follows: (1) being Italian; (2) aged between 18 and 65 years; (3) having a BMI ≥ 30. The exclusion criteria included any form of psychiatric diseases and/or mental impairments that could compromise the understanding of the questionnaires. After being informed about the research and obtaining written informed consent to participate, the participants were asked to provide their socio-demographic data and complete self-report questionnaires. The study was approved by the Territorial Ethical Committee (CET 5), Lombardy Region, Italy (approval number: 205/24; date of approval: 23 April 2024; research code: 01C415; acronym: METAOBES). The research was conducted in accordance with the Declaration of Helsinki and its subsequent revisions.

### 2.2. Measures

The internal medical staff measured weight and height. Standing height was determined by using a Harpenden Stadiometer (Holtain Limited, Crymych, Dyfed, UK). Weight was measured to the nearest 0.1 kg using an electronic scale (RoWU 150, Wunder Sa.bi., Trezzo sull’Adda, Italy). Body mass index (BMI) was calculated according to the proper formula: kg/m^2^. Demographic data were collected by asking the participants to complete a self-report form. Clinical data about worry, rumination, emotional dysregulation, and emotional eating were obtained using the following questionnaires:

The Penn State Worry Questionnaire (PSWQ) [29] is a widely used self-report measure designed to assess the tendency to engage in excessive and uncontrollable worry. The questionnaire consists of 16 items (i.e., “*I worry all the time*”), rated on a 5-point Likert scale ranging from 1 (“Not at all typical of me”) to 5 (“Very typical of me”). Higher scores indicate greater worry severity. The PSWQ score ranges from 16 to 80. The PSWQ has demonstrated strong internal consistency, test–retest reliability, and validity. For the Italian population, the PSWQ was validated by Morani et al. [30], confirming its psychometric properties, including high internal consistency and good convergent validity with measures of anxiety and worry. The Italian version maintains the factorial structure of the original scale. In our sample, the internal consistency of the PSWQ was very good (α = 0.871).

The Ruminative Response Scale (RRS) [31] is a widely used self-report questionnaire designed to assess rumination, a maladaptive cognitive process characterized by a repetitive and passive focus on one’s distress and its possible causes and consequences. The RRS is composed of 22 items (i.e., “*I think about how alone I feel”*) rated on a Likert scale ranging from 1 (“Almost never”) to 4 (“Almost always”), with higher scores indicating greater levels of depressive ruminative thinking. The RRS score ranges from 22 to 88. For this study, we used the Italian-validated version of the RRS [32], which demonstrated good psychometric properties, including internal consistency and construct validity. In our sample, the internal consistency of the RRS was excellent (α = 0.938).

The Anger Rumination Scale (ARS) [33] is a self-report questionnaire designed to assess the tendency to engage in repetitive and prolonged thoughts about anger-eliciting experiences. The ARS consists of 19 items (i.e., “*I re-enact the anger episode in my mind after it has happened”*), rated on a Likert scale from 1 (“Almost never”) to 4 (“Almost always”), with higher scores indicating a greater propensity for anger rumination. The ARS score ranges from 19 to 76. For this study, we used the Italian-validated version of the ARS [34], which has demonstrated good psychometric properties, including internal consistency and construct validity. In our sample, the internal consistency of the ARS was excellent (α = 0.924).

The Difficulties in Emotion Regulation Scale (DERS) [7] is a self-report instrument developed to assess multiple aspects of emotional dysregulation. It comprises 36 items (i.e., “*When I’m upset, I feel guilty for feeling that way*”), each rated on a 5-point Likert scale ranging from 1 (“Almost never”) to 5 (“Almost always”). Higher scores indicate greater emotional dysregulation. The DERS score ranges from 36 to 180. The Italian adaptation of the DERS [35] has been validated to ensure cultural relevance and psychometric robustness. The translated version maintains the original 36-item structure and six-factor model. Psychometric evaluations demonstrated adequate internal consistency, with Cronbach’s alpha coefficients comparable to the original scale. In our sample, the internal consistency of the DERS was very good to excellent (α = 0.899).

The Dutch Eating Behavior Questionnaire (DEBQ) [13] is a widely used self-report instrument designed to assess various eating behaviors, including restrained eating, emotional eating, and external eating. Among its three subscales, the Emotional Eating subscale (EE-DEBQ) specifically measures the tendency to eat in response to negative emotional states such as anxiety, sadness, or stress. It consists of 13 items, each rated on a 5-point Likert scale, from 1 (“Never”) to 5 (“Very often”), where respondents indicate how frequently they engage in emotional eating behaviors (i.e., “*Do you have a desire to eat when you are nervous*?”). Scores are calculated as means (score range: from 1 to 5). Higher scores indicate greater emotional eating. The Italian version of the DEBQ [36] demonstrated psychometric properties comparable to those of the original instrument. In our sample, the internal consistency of the EE-DEBQ was excellent (α = 0.960).

### 2.3. Statistical Analysis

The required sample size for this mediation analysis was determined using G*Power 3.1. The study examines the indirect effect of Difficulties in Emotion Regulation (DERS) on Emotional Eating (EE-DEBQ) through three mediators: the Penn State Worry Questionnaire (PSWQ), Ruminative Response Scale (RRS), and Anger Rumination Scale (ARS). Given that the mediation model involves multiple regression pathways (predictor → mediator; mediator → dependent variable), power analysis was conducted using the linear multiple regression: fixed model, R^2^ deviation from zero approach, and fixing a large effect size (f^2^ = 0.35), a significance level of α = 0.05, and a statistical power of 0.80. Based on these parameters, the estimated required sample size was approximately 80–100 participants.

Descriptive statistics, including means, standard deviations, frequencies, and percentages (as appropriate for the nature of the variables), were computed to summarize the sample characteristics. The normal distribution of the variables was assessed using skewness and kurtosis indices. Pearson’s correlation coefficients were computed to examine the relationships between the variables of interest, in particular, to determine the strength and direction of associations between DERS, PSWQ, RRS, ARS, and EE-DEBQ. Additionally, possible gender differences in DERS, PSWQ, RRS, ARS, and EE-DEBQ were assessed using independent sample *t*-tests. Significance levels were set at *p* < 0.05.

Three mediation models were tested to examine the relationship between difficulties in emotional regulation (DERS) as a predictor, worry (PSWQ), rumination (RRS and ARS) as separate mediators, and emotional eating (EE-DEBQ) as the dependent variable. Mediation analyses were conducted employing a bootstrapping method [37] with 5.000 resamples to estimate indirect effects. Standardized regression coefficients were calculated for all paths, including the direct effect of DERS on EE-DEBQ and the indirect effects through the proposed mediators. The statistical significance of mediation effects was determined by examining the bias-corrected 95% confidence intervals, with mediation considered significant if the intervals did not include zero. Although conducting multiple analyses increases the risk of Type I error, separate models were tested due to the high correlations among the mediators and the limited sample size, which did not allow for a more complex model with multiple mediators simultaneously. This approach enabled us to observe the individual effect of each mediator on the outcome. Analyses were performed using Jamovi (version 2.6).

## 3. Results

The sample was composed of ninety hospitalized Italian adults with obesity (53 males, mean age ± SD: 49.1 ± 11; body mass index (BMI): 47.9 ± 10.6: kg/m^2^; 37 females, mean age ± SD: 52.9 ± 10.6 years, mean body mass index (BMI): 44.3 ± 6.9: kg/m^2^). Most of them lived in Northern Italy (85.6%), had a high school degree (56.7%), were single (46.1%), and were employed (41.6%). The demographic variables considered did not influence the study’s results. Descriptive statistics are shown in Table 1.

Correlations between the study variables showed a strong positive correlation between worry (PSWQ) and rumination (RRS) (r = 0.667, *p* < 0.001), indicating that individuals with higher levels of worry tend to engage in more ruminative thinking. Similarly, worry (PSWQ) was significantly correlated with anger rumination (ARS: r = 0.608, *p* < 0.001), emotional dysregulation (DERS: r = 0.516, *p* < 0.001), and emotional eating (EE-DEBQ: r = 0.471, *p* < 0.001). Rumination (RRS) was strongly correlated with emotional dysregulation (DERS; r = 0.745, *p* < 0.001) and anger rumination (ARS; r = 0.633, *p* < 0.001). Additionally, emotional eating (EE-DEBQ) was significantly correlated with rumination (RRS; r = 0.548, *p* < 0.001), anger rumination (ARS; r = 0.426, *p* < 0.001), and emotional dysregulation (DERS; r = 0.355, *p* < 0.001).

The analysis of gender differences revealed statistically significant higher scores for worry (PSWQ: *p* = 0.041), rumination (RRS: *p* = 0.011), and emotional eating (DEBQ: *p* = 0.002) in females than in males. No significant differences were found in anger rumination (ARS) and emotional dysregulation (DERS). The results are shown in Table 2.

**Figure 1:**
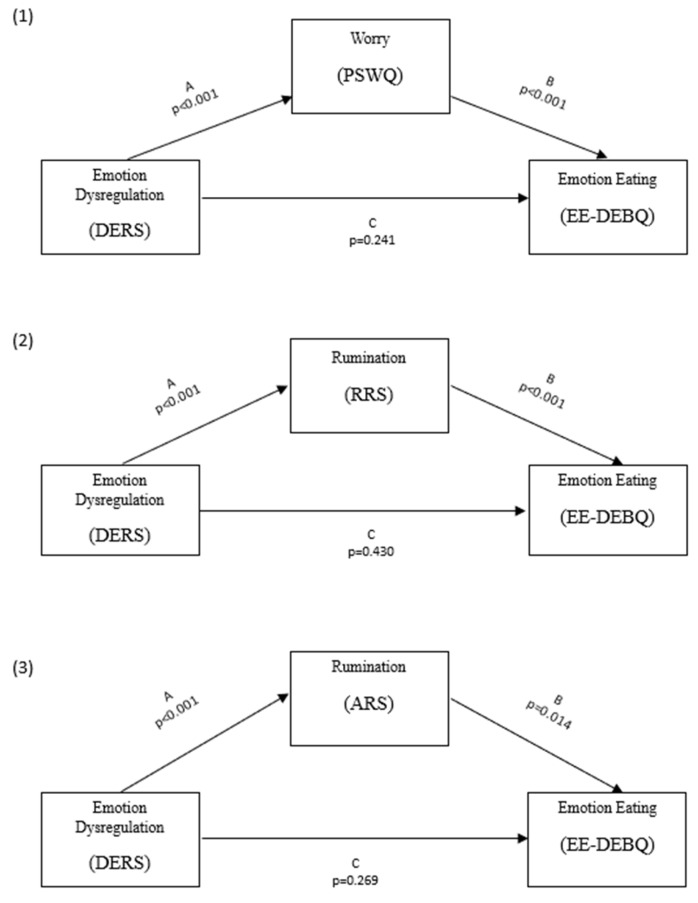
(1) Graphical description of the mediation model with DERS as predictor, PSWQ as mediator, and EE-DEBQ as dependent variable; (2) Graphical description of the mediation model with DERS as predictor, RRS as mediator, and EE-DEBQ as dependent variable; (3) Graphical description of the mediation model with DERS as predictor, ARS as mediator, and EE-DEBQ as dependent variable.

The mediation analyses revealed significant indirect effects across all the models, suggesting the presence of mediation effects of worry (PSWQ) and rumination (RRS and ARS) in the relationships between emotional dysregulation (DERS) and emotional eating (EE-DEBQ). In particular, for the PSWQ model (1), the indirect effect was significant (β = 0.010, SE = 0.003, *p* = 0.002), accounting for 57.1% of the total effect (β = 0.018, SE = 0.006, *p* = 0.004), whereas the direct effect was not significant (β = 0.008, SE = 0.006, *p* = 0.229). Similarly, in the RRS model (2), the indirect effect was significant (β = 0.025, SE = 0.005, *p* < 0.001), explaining 79.7% of the total effect (β = 0.018, SE = 0.006, *p* = 0.006), while the direct effect was non-significant (β = −0.006, SE = 0.008, *p* = 0.30). Lastly, the ARS model (3) also exhibited a significant indirect effect (β = 0.010, SE = 0.004, *p* = 0.019), constituting 55.5% of the total effect (β = 0.018, SE = 0.006, *p* = 0.004), with the direct effect remaining non-significant (β = 0.008, SE = 0.007, *p* = 0.273). These findings highlight the critical mediating role of worry and rumination that drive the observed relationships between DERS and EE-DEBQ. A graphical depiction of the models is presented in Figure 1.

Given the significant differences between males and females in PSWQ, RRS, and EE-DEBQ, an additional moderating effect of gender on the mediation pathways between DERS (predictor), PSWQ, RRS, and ARS (mediators), and EE-DEBQ (outcome) was tested. No significant indirect effects were found, suggesting that gender did not significantly moderate the explored relationships.

## 4. Discussion

The current study examined the mediating role of worry and rumination in the relationships between difficulties in emotional regulation and emotional eating among hospitalized adults with obesity. More specifically, we initially explored the relationships between the study variables to verify whether the basic assumptions for mediation were met. In this regard, the results highlighted significant and positive correlations between worry, rumination, emotional dysregulation, and emotional eating. These results were in line with previous research, which suggested that emotional dysregulation is associated with a tendency to engage in repetitive negative thinking (worry and rumination) and metacognitive beliefs [38]. Then, we also preliminarily explored possible gender differences among the variables, revealing higher scores for worry, rumination, and emotional eating in females than in males, while no significant differences were found for anger rumination and emotional dysregulation. These results aligned with prior studies that found gender differences for both worry and rumination, with women generally exhibiting higher levels of these cognitive processes than men [39,40] and differences in eating behaviors, with women reporting significantly higher levels of eating pathology [41].

Once the preliminary analyses were explored, we investigated whether difficulties in emotional regulation predicted emotional eating, with worry and rumination serving as separate mediators. Our results showed that there were significant indirect effects across all models, thus suggesting the presence of mediation effects of worry (PSWQ) and rumination (RRS and ARS) in the relationships between emotional dysregulation (DERS) and emotional eating (EE-DEBQ). These results indicated that emotional dysregulation may contribute to emotional eating behaviors, indirectly increasing perseverative negative thinking patterns, specifically worry and rumination. This result is in line with previous studies that have identified worry and rumination as key transdiagnostic processes linking emotional distress to maladaptive behaviors. In this respect, Aldao and colleagues [26] highlighted that rumination was strongly associated with a range of maladaptive emotional regulation strategies and was a common risk factor across several forms of psychopathology, including disordered eating.

Given the gender-related differences in worry, rumination, and emotional eating, we also explored the possible moderating effect of gender in the mediation pathways between emotional dysregulation, worry, rumination, and emotional eating. No significant indirect effects were found, suggesting that gender does not significantly moderate the explored relationships. Although the results indicated that women reported higher levels of emotional eating compared to men, the moderation analysis did not reveal a significant moderating effect of gender on the relationship between worry and/or rumination and emotional eating. This finding suggests that while gender differences exist in the overall levels of emotional eating, the strength and direction of the associations between worry and rumination and emotional eating appear to be consistent across the genders. Therefore, the mechanisms linking these psychological processes to emotional eating may operate similarly in both men and women.

From a clinical standpoint, the findings of the present study have important implications. Cognitive Behavioral Therapy (CBT) [42]—a gold standard treatment for obesity.

From a clinical standpoint, the findings of the present study have important implications. Cognitive Behavioral Therapy (CBT)—a gold standard treatment for obesity [43,44]—has traditionally focused on addressing dysfunctional eating patterns and promoting behavior change [43]. However, incorporating modules that target cognitive-emotional processes such as worry and rumination may enhance the treatment effectiveness. Interventions such as metacognitive therapy could be adapted to reduce repetitive negative thinking and improve emotional regulation, ultimately supporting healthier eating behaviors and weight management [45,46]. In this context, studying the mediating role of worry and rumination in the link between emotion dysregulation and obesity is not only theoretically significant but also clinically valuable. It may inform the development of integrative psychological interventions tailored to the cognitive-affective profiles of individuals with obesity.

Several limitations should, however, be acknowledged. First, the cross-sectional design of the study limits our ability to infer causality regarding the directionality of the relationships between emotional dysregulation, worry, rumination, and emotional eating. Second, the use of only self-report measures may have introduced response biases that could affect the validity of the findings. Third, the sample consisted exclusively of hospitalized individuals with obesity seeking a 3-week multidisciplinary metabolic rehabilitation, which limits the generalizability of the results to the broader population. A non-obese, age- and gender-matched control group would allow useful comparisons that could clarify whether the observed mechanisms are specific to obesity-related emotional eating. Finally, the sample size, although adequate for the mediation analysis conducted, may limit the statistical power to detect smaller effects or interactions, particularly in subgroup analyses. Future studies should consider incorporating multi-method assessments, including behavioral or physiological indicators of emotional regulation and eating behavior, longitudinal measures, and a normal-weight, age- and gender-matched control group.

## Figures and Tables

**Table 1:** Descriptive statistics of the sample.

		*n* (%)	*M (SD)*
Gender	Males	53 (58.9%)	
	Females	37 (41.1%)	
Age			50.8 (10.9)
BMI			46.4 (9.40)
Region of origin-Italy	Northern	77 (85.6%)	
	Central	3 (3.3%)	
	Southern	10 (11.1%)	
Marital status	Single	41 (46.1)	
	Cohabiting	30 (33.7%)	
	Married	14 (15.7%)	
	Separated/divorced	5 (4.5%)	
Educational level	Middle school	28 (31.1%)	
	High school	51 (56.7%)	
	Bachelor’s degree	11 (12.2%)	
Occupation	Employed	37 (41.6%)	
	Self-employed	18 (20.2%)	
	Retired	9 (10.1%)	
	Unemployed	20 (22.5)	
	Other	6 (5.6%)	
DERS			84.81 (20.26)
PSWQ			45.22 (12.17)
RRS			38.82 (12.39)
ARS			29.92 (9.01)
EE-DEBQ			1.49 (1.08)

Notes: DERS: Difficulties in Emotion Regulation Scale; PSWQ: Penn State Worry Questionnaire: RRS: Ruminative Response Scale; ARS: Anger Rumination Scale: EE-DEBQ: Emotional Eating subscale of the Dutch Eating Behavior Questionnaire.

**Table 2:** Comparisons between males and females in all the study variables.

	Males (*n* = 53) *M (SD)*	Females (*n* = 37) *M (SD)*	*t* (*p*)	Cohen’s *d* Effect Size
DERS	81.6 (20.35)	89.4 (19.52)	−1.80 (0.076)	−0.385
PSWQ	43.0 (11.21)	48.4 (12.95)	−2.08 (0.041) *	−0.445
RRS	36.1 (11.24)	42.8 (13.05)	−2.61 (0.011) *	−0.560
ARS	25.9 (8.67)	28.4 (9.43)	−1.26 (0.211)	−0.270
EE-DEBQ	1.20 (0.95)	1.90 (1.12)	−3.15 (0.002) *	−0.675

Notes: DERS: Difficulties in Emotion Regulation Scale; PSWQ: Penn State Worry Questionnaire: RRS: Ruminative Response Scale; ARS: Anger Rumination Scale: EE-DEBQ: Emotional Eating subscale of the Dutch Eating Behavior Questionnaire. * *p* < 0.05.

## Data Availability

Raw data will be uploaded on www.Zenodo.org immediately after the acceptance of the manuscript, and they will be available upon a reasonable request to the authors A.G.U. and A.S.
